# Supplementary material for: Sorting at embryonic boundaries requires high heterotypic interfacial tension
Source: Nat Commun. 2017 Jul 31;8:157. doi: 10.1038/s41467-017-00146-x (PMC5537356; doi:10.1038/s41467-017-00146-x)
Supplement: Supplementary file 2 — Supplementary Software 1 [file 41467_2017_146_MOESM2_ESM.zip › PottsModel/SrcPottsModel/doc/utils/package-use.html]

Uses of Package utils


JavaScript is disabled on your browser.


Skip navigation links


- Overview
- Package
- Class
- Use
- Tree
- Deprecated
- Index
- Help

- Prev
- Next

- Frames
- No Frames

- All Classes

# Uses of Package utils

- Packages that use utils

  | Package | Description |
  | --- | --- |
  |  |  |
  | --- | --- |
  | utils |  |
- Classes in utils used by utils

  Class and Description

  HSLColor

  The HSLColor class provides methods to manipulate HSL (Hue, Saturation
  Luminance) values to create a corresponding Color object using the RGB
  ColorSpace.

Skip navigation links


- Overview
- Package
- Class
- Use
- Tree
- Deprecated
- Index
- Help

- Prev
- Next

- Frames
- No Frames

- All Classes
